# Immunological evaluation of patients with mucopolysaccharidosis (MPS)

**DOI:** 10.1186/1939-4551-8-S1-A158

**Published:** 2015-04-08

**Authors:** Carolina Aranda, Ana Maria Martins, Marcia Carvalho Mallozi, Beatriz Costa Carvalho, Dirceu Sole

**Affiliations:** 1 Hospital Do Servidor Publico Municipal De São Paulo, Brazil; 2 Pediatrics. Unifesp, Brazil; 3 Federal University of Sao Paulo, Brazil; 4 Brazilian Society, Brazil

## Background

MPS is a group of metabolic diseases caused by deficiency of lysosomal enzymes that degrade glycosaminoglycans (GAG). Recurrent respiratory infections, sleep disturbances, upper and lower airway obstruction are frequently reported in MPS’ patients. However, cellular accumulation of GAG fragments leading to progressive multi-system manifestations can clutter homeostasis also modify the function of other cellular components, their signals and produce substances. The aim of study is to evaluate immunologically MPS’ patients to clarify why they are prone to infections.

## Methods

Eighteen MPS patients (mean age = 13 yr-old, from 5 to 32 years) in enzyme replacement therapy (ERT), 88% male were evaluated (type I = 5, type II = 9 and type VI = 4) by measurement of complete blood count (CBC) and quantitative/ qualitative serum immunoglobulins (Ig; G, M and A) and review of their immunization schedules (BCG, hepatitis B and rubella).

## Results

All patients had previous history of wheezing and pneumonia that had significant improvement after initiation of ERT. Only one patient had iron deficiency anemia. Two patients had neutrophils lower than expected and all patients had adequate number of lymphocytes. All patients were vaccinated for BCG, however one patient had lymphnode tuberculosis. Only one patient had IgG serum levels lower than 3^rd^ percentile. Three patients had IgM levels 3^rd^lower percentile. Despite complete hepatitis B vaccination schedule, 10 (55%) patients showed absence of response to vaccine; and 1 patient (5.5%) showed no response to rubella vaccine .

## Conclusions

The immunological evaluation of MPS patients is mandatory, especially for the high frequency of respiratory infections presented by them. More studies on the humoral and innate immunity are needed to understand the disease and improve the treatment of these complications.

